# Dietary characteristics of community-dwelling older adults with poor appetite: a cross-sectional analysis

**DOI:** 10.1093/ageing/afae040

**Published:** 2024-05-15

**Authors:** Pia Scheufele, Anja Rappl, Marjolein Visser, Eva Kiesswetter, Dorothee Volkert

**Affiliations:** Institute for Biomedicine of Ageing, Faculty of Medicine, Friedrich-Alexander-Universität Erlangen-Nürnberg, Nuremberg, Germany; Institute of Medical Informatics, Biometry and Epidemiology, Faculty of Medicine, Friedrich-Alexander-Universität Erlangen-Nürnberg, Erlangen, Germany; Department of Health Sciences, Amsterdam Public Health Research Institute, Vrije Universiteit Amsterdam, Amsterdam, The Netherlands; Institute for Biomedicine of Ageing, Faculty of Medicine, Friedrich-Alexander-Universität Erlangen-Nürnberg, Nuremberg, Germany; Institute for Evidence in Medicine, Faculty of Medicine and Medical Center, University of Freiburg, Freiburg, Germany; Institute for Biomedicine of Ageing, Faculty of Medicine, Friedrich-Alexander-Universität Erlangen-Nürnberg, Nuremberg, Germany

**Keywords:** older adults, anorexia of ageing, poor appetite, malnutrition, diet quality

## Abstract

**Rationale:**

Poor appetite is considered a key factor in the development of malnutrition, a link that can be explained by alterations in dietary intake. Given the limited data on dietary characteristics in community-dwelling older adults with poor appetite, the present study aimed to examine whether poor appetite is associated with lower nutrient intake and more unfavourable food choices.

**Methods:**

In 569 participants of the Longitudinal Aging Study Amsterdam aged ≥70 years appetite was assessed using the Simplified Nutritional Appetite Questionnaire and dichotomised into normal (>14) and poor (≤14). Intake of energy, 19 nutrients, 15 food groups, the Dutch Healthy Diet Index 2015 (DHD15) and Mediterranean Diet Score (MDS) were calculated from a food frequency questionnaire. Dietary differences between appetite groups were examined using Mann–Whitney *U* test and binary logistic regression adjusted for potential confounders.

**Results:**

Mean age was 78 ± 6 years and 52% were female. Appetite was poor in 12.5% of participants. Energy intake was 1951 (median; quartiles 1–3: 1,653–2,384) kcal/day with no difference between appetite groups. Poor appetite was associated with lower intake of protein (OR 0.948, 95%CI 0.922–0.973), folate (0.981, 0.973–0.989), zinc (0.619, 0.454–0.846), vegetables (0.988, 0.982–0.994) and lower scores of DHD15 (0.964, 0.945–0.983) and MDS (0.904, 0.850–0.961), as well as higher intake of carbohydrates (1.015, 1.006–1.023), and vitamins B_2_ (4.577, 1.650–12.694) and C (1.013, 1.005–1.021).

**Conclusions:**

Community-dwelling older adults with poor appetite showed poorer diet quality with a lower intake of protein, folate, zinc and vegetables, compared with those reporting normal appetite and should be advised accordingly.

## Key Points

- Evidence on dietary characteristics of community-dwelling older adults with poor appetite at risk of malnutrition is limited.
- Previous multivariate analyses suggest a lower intake of protein and fibre but are inconsistent regarding energy intake.
- The current analysis uses European data with extensive information on macro-, micronutrients, food groups and diet quality.
- Poor appetite was associated with lower diet quality and lower intake of protein, folate, zinc and vegetables.
- Poor appetite was also associated with higher carbohydrate, and vitamins B_2_ and C intake without a difference in energy intake.

## Introduction

Because of the central role of appetite as a driver of food intake, poor appetite is considered a key factor in the development of malnutrition [1]. Poor appetite is reported in up to 27% of community-dwelling older adults [1–4] and is associated with adverse health outcomes such as disability [5] and mortality [6, 7]. The relationship between appetite and malnutrition is primarily influenced by diet, and it can be assumed that people with poor appetite consume less food and may also make less favourable food choices. At present, knowledge about the dietary characteristics of older people with poor appetite is limited.

Although appetite is influenced by various factors, such as chewing difficulties or depression [8, 9], only two studies to date have taken these influencing factors into account in multivariate analyses [3, 10]. van der Meij *et al*. [3] analysed cross-sectional data from the Health, Aging, and Body Composition study to investigate associations between poor appetite and energy, macronutrient and food group intake amongst 2,597 community-dwelling adults aged between 70 and 79 years in the USA. When compared with very good appetite, those with poor appetite had a lower intake of protein, fibre, solid foods, fruits, vegetables, grains and protein-rich foods (meat, fish, beans, egg) as well as a higher energy density of solid foods and a higher intake of fats, milk (products), sweets or sodas. In a smaller study, Hara *et al*. [10] examined the relationship between poor appetite and inadequate energy, macronutrient and mineral intake in 130 adults aged 60 years or older who received outpatient care in Brazil. Similar to the findings of van der Meij *et al*. [3], poor appetite was associated with an inadequate intake of protein and fibre and in addition with an inadequate intake of energy, iron and zinc [10].

A better understanding of the diet in this group could help identify relevant dietary characteristics to provide approaches for targeted nutritional counselling and contribute to the prevention of malnutrition. Given the very limited data available, the present study therefore aimed to conduct a detailed analysis to examine whether poor appetite is associated with lower nutrient intake and more unfavourable food choices.

## Methods

### Study design and data source

For the present analyses, data from the Longitudinal Aging Study Amsterdam (LASA), a prospective cohort study in community-dwelling older adults in the Netherlands, were used. The study started in 1992/93 with a nationally representative sample of adults aged 55–85 years and is still ongoing. In addition to the original cohort (*n*(I) = 3,805), two further cohorts aged 55–65 years (*n*(II) = 1,002, *n*(III) = 1,023) were recruited in 2002/03 (II) and 2012/13 (III). Data on different topics (biomaterial, care, physical functioning, emotional functioning, cognitive functioning, social functioning, demographics and work) were collected every three years in the main study to investigate the determinants and consequences of ageing. Details of the LASA main study are described elsewhere [11]. In 2014/15, the Nutrition and Food-related Behaviour study was conducted amongst a subsample of the LASA participants. Out of the 2,331 participants of measurement waves 2011/12 (cohorts I, II) and 2012/13 (cohort III), 1,439 participated in this ancillary study. The study consisted of a self-completed questionnaire on food-related behaviour and a semi-quantitative food frequency questionnaire (FFQ).

### Study population

Of the participants in the Nutrition and Food-related Behaviour study, 844 were excluded from the present study because of their age below 70 years [9, 12]. Additionally, 17 participants were excluded because of incomplete FFQ data and nine because of implausible low (two men < 800 kcal, one woman < 500 kcal) or high (four men > 4,000 kcal, two women > 3,500 kcal) energy intake per day [13]. Thus, 569 participants were included in the analysis.

### Appetite

Appetite was assessed using the Simplified Nutritional Appetite Questionnaire (SNAQ), which was part of the questionnaire on food-related behaviour. SNAQ is a four-item instrument asking for appetite, satiety, taste of food and meal frequency per day, which is validated in community-dwelling older adults [14]. The SNAQ score is calculated from the answers (each 1–5 points) of the four items and ranges from 4 to 20. A score from 4 to 14 is considered as an indicator of having poor appetite; 15–20 indicates a normal appetite [14].

### Dietary characteristics

Food intake was assessed between 2014 and 2015 using the Dutch version of the FFQ from the Healthy Life in an Urban Setting study [15], adapted from an existing validated FFQ [16]. The FFQ asks for the consumption of 238 food items in the past four weeks. Average daily intake of the following nutrients was calculated by linking each food item to one or more foods of a nutrient database based on the Dutch Food Composition Table 2011 [17]: macronutrients (carbohydrates, protein (animal, plant), fat, fibre, alcohol, water), vitamins (A, B_1_, B_2_, B_6_, B_12_, C, D, folate), minerals (calcium, iron, phosphorus, magnesium, zinc). Total daily energy intake was calculated by summing the calories of all foods consumed per day. Daily intake of the following food groups was calculated from the sum of the consumed amount of all food items in the respective category: rice/pasta, bread, potatoes/other tubers, legumes, vegetables, fruits, dairy products except cheese, cheese, eggs, total meat/meat products, (shell) fish, sugar/confectionery, cakes/sweet baked goods, non-alcoholic beverages and alcoholic beverages.

Two diet quality indices were calculated. The Mediterranean Diet Score (MDS) is a measure of adherence to the traditional Mediterranean diet, calculated by summing the scores from 0 to 5 based on the daily or weekly consumption of eleven food groups (fruit, vegetables, legumes, potatoes, non-refined cereals, red meat and products, poultry, fish, olive oil, full-fat dairy products, alcoholic beverages). The total score ranges from 0 to 55, with a higher score indicating a higher adherence [18]. The Dutch Healthy Diet Index 2015 (DHD15) reflects adherence to the Dutch dietary guidelines of 2015. The DHD15 index is a sum score composed of the individual scores from 0 to 10 of 15 food groups (vegetables, fruit, whole grain products, legumes, nuts, dairy, fish, tea, fats and oils, coffee, red meat, processed meat, sweetened beverages, and fruit juices, alcohol, salt) and ranges from 0 to 150, with a higher score indicating a higher adherence [19]. As the FFQ in LASA did not collect data on the consumption of salt and the type of coffee consumed, these components were omitted and the score was consequently based on 13 components, resulting in a score of 0–130.

### Participants’ general characteristics

Participants’ general characteristics were obtained during the main measurement wave of 2011/12 as part of the main interview (age, sex, educational status, multimorbidity, physical performance, self-rated health, depression, cognitive impairment), and the medical interview (measured body weight (BW) and height, any unintentional weight loss in past 6 months, medication use) or by a self-administered questionnaire (eating less because of chewing problems, mostly eating alone).

Educational status was categorised into high (university, college or higher vocational education), middle (general secondary education, intermediate and lower vocational education and general intermediate education) and low (elementary education completed or not completed). Multimorbidity referred to the presence of at least two of seven major chronic conditions (cardiac disease, peripheral atherosclerosis, stroke, diabetes mellitus, obstructive lung disease, arthritis, cancer). Physical performance was assessed using the validated Short Physical Performance Battery (SPPB) consisting of a chair stand, tandem stand and walk test. Each test was scored from 0 to 4, resulting in a sum score of 0 to 12. A SPPB score of ≤8 points was considered a low physical performance [20]. Polypharmacy was defined as the use of ≥5 different medicines in the past 2 weeks. Self-perceived health was obtained by the question ‘How do you rate your health in general?’, which had five answer categories 1 ‘excellent’, 2 ‘good’, 3 ‘fair’, 4 ‘sometimes good/sometimes poor’ and 5 ‘poor’. Depression was indicated by a Center for Epidemiologic Studies Depression Scale (CES-D) score of ≥16 (out of 60) [21, 22]. Cognitive functioning was assessed using the Mini-Mental State Examination (MMSE) [23], with a score of ≤23 (out of 30) defined as a poor cognitive status [24]. Body mass index (BMI) was calculated by dividing the BW in kilogrammes by the height in metres squared.

### Statistical analyses

Continuous variables are described by median with interquartile range, age is additionally described by mean and standard deviation; categorical variables are reported as relative frequencies, both according to appetite group. Differences between participants with poor and normal appetite were tested using Mann–Whitney *U* test or Pearson’s chi-square test. A *P* value of <0.05 was considered a statistically significant result for group comparison.

Associations between dietary characteristics (continuous variables) and appetite were further assessed using binary logistic regression analyses (dependent variable: appetite). Because of the small proportion of persons with poor appetite in the sample, the outcome is unbalanced and thus the number of explanatory covariates the logistic regression model could tolerate was limited. Therefore, to achieve results and avoid non-convergence of the model’s algorithm, we calculated several logistic regression models with a restricted number of explanatory covariates and subsequently compared them. All models were adjusted for variables that were found to be associated with poor appetite in a previous analysis [9], namely, sex, any unintended weight loss in past 6 months, eating less because of chewing problems, polypharmacy, depression and additionally for age, educational status and energy intake. In order to make the comparison viable, Bonferroni correction was applied to the hypothesis tests of the ensuing regression coefficients and an adjusted *P* value of <0.004 was considered a statistically significant result. Separate models were performed for the intake of energy, each macronutrient and each of the diet quality indices. Vitamins, minerals and food groups were combined in one model, respectively. Complete case analyses were performed excluding cases with missing data in the characteristics used for adjusting the models. Statistical analyses were performed using SPSS version 26.0 (IBM, Munich, Germany).

## Results

### Prevalence of poor appetite and participants’ general characteristics

The prevalence of poor appetite was 12.5% (*n* = 71). The mean age of the total sample was 78 ± 6 years, 52% was female. Participants with poor appetite were significantly older, had lower educational status and reported eating less because of chewing problems more often than participants with normal appetite. In addition, multimorbidity, polypharmacy, poor health, depression, mostly eating alone and low physical performance were more prevalent in participants with poor appetite (Table 1).

**Table 1:** General characteristics of the 569 participants aged 70 years and older of the Nutrition and Food-related Behaviour ancillary study of the LASA, stratified by appetite status

Variable		Normal appetite (*n* = 498)	Poor appetite (*n* = 71)	*P* value
**Age, mean (SD);** **median (Q1**–**Q3) [years]**		77.6 (6.0); 75.9 (72.8–81.2)	81.5 (6.6); 81.8 (75.8–85.8)	<0.001
**Men [%]**		48.8	39.4	0.163
**Educational status [%]**	Low	15.1	39.4	<0.001
	Middle	58.6	50.7	
	High	26.3	9.9	
**BMI, median (Q1**–**Q3) [kg/m**^**2**^**]**		27.0 (24.8–29.6)	26.7 (24.9–30.7)	0.608
	*Missing, %*	*3.6*	*9.9*	
**Any involuntary weight loss in past 6 months [%]**		6.2	9.9	0.202
	*Missing*	*3.4*	*7.0*	
**Eating less** **because of** **chewing problems [%]**		0.8	7.0	0.002
	*Missing*	*2.0*	*5.6*	
**Multimorbidity [%]**		40.4	57.7	0.007
**Low physical performance [%]** (SPPB ≤ 8)		43.6	60.6	0.002
	*Missing*	*5.0*	*9.9*	
**Polypharmacy [%]**		27.3	39.4	0.022
	*Missing*	*3.0*	*7.0*	
**Self-rated health [%]**	Excellent	11.6	11.3	<0.001
	Good	62.0	35.2	
	Fair	20.1	28.2	
	Sometimes good/poor	4.8	19.7	
	Poor	1.2	5.6	
	*Missing*	*0.2*	*0.0*	
**Depression [%]** (CES-D ≥ 16)		8.0	25.4	<0.001
	*Missing*	*0.2*	*0.0*	
**Mostly eating alone [%]**		27.5	49.3	<0.001
	*Missing*	*2.0*	*4.2*	
**Poor cognitive status [%]** (MMSE ≤ 23)		1.2	4.2	0.090

Poor appetite is defined as SNAQ ≤ 14. Comparison between participants with normal and poor appetite: Pearson’s chi-square test (categorical variables), Mann–Whitney *U* test (continuous variables). A *P* value of <0.05 was considered statistically significant. Quartiles 1–3 (Q1–Q3).

### Dietary characteristics

#### Intake of energy and nutrients

Median daily energy intake was 2,211 (1,852–2,544) kcal in males and 1,810 (1,539–2,106) kcal in females. Energy intake of the total sample, as well as within the group of men and the group of women, did not differ between the appetite groups.

Table 2 shows the median daily nutrient intake of participants with normal and poor appetite. Participants with poor appetite had a lower intake of total, animal and plant protein, fibre, alcohol, vitamin B_6_, B_12_, folate, calcium, iron, phosphorus, magnesium and zinc than those with normal appetite based on unadjusted comparisons.

**Table 2:** Energy and nutrient intake per day of the 569 participants aged 70 years and older of the Nutrition and Food-related Behaviour ancillary study of the LASA, stratified by appetite (median and quartiles 1–3)

Variable		Normal appetite (*n* = 498)	Poor appetite (*n* = 71)	*P* value
**Energy [kcal/d]**		1,966 (1,665–2,391)	1,885 (1,468–2,366)	0.123
**Macronutrients [g/d]**	Carbohydrates	207 (172–249)	218 (169–268)	0.409
	Protein	75 (64–90)	62 (52–81)	<0.001
	Animal protein	46 (37–58)	36 (29–50)	<0.001
	Plant protein	29 (24–36)	24 (21–32)	<0.001
	Fat	70 (56–92)	67 (47–86)	0.152
	Fibre	22 (18–26)	18 (15–25)	<0.001
	Alcohol	6.7 (0.4–18.4)	0.6 (0.0–10.7)	<0.001
	Water	2,315 (1,911–2,698)	2,143 (1,662–2,528)	0.010
**Vitamins**	Vitamin A (RAE) [μg/d]	817 (604–1,097)	736 (563–1,051)	0.223
	Vitamin B_1_ [mg/d]	1.0 (0.8–1.2)	0.9 (0.7–1.2)	0.096
	Vitamin B_2_ [mg/d]	1.5 (1.2–1.7)	1.2 (1.0–1.7)	0.086
	Vitamin B_6_ [mg/d]	1.6 (1.3–2.0)	1.5 (1.1–1.9)	0.018
	Vitamin B_12_ [μg/d]	4.5 (3.5–6.2)	3.6 (2.7–5.3)	<0.001
	Vitamin C [mg/d]	127 (92–169)	123 (65–186)	0.150
	Vitamin D [μg/d]	3.6 (2.6–4.9)	3.1 (2.2–4.4)	0.055
	Folate (FE) [μg/d]	275 (227–333)	226 (188–291)	<0.001
**Minerals [mg/d]**	Calcium	963 (767–1,246)	838 (621–1,063)	0.003
	Iron	10 (9–12)	9 (7–11)	<0.001
	Magnesium	343 (290–395)	296 (242–357)	<0.001
	Phosphorus	1,444 (1,204–1,696)	1,212 (1,006–1,566)	<0.001
	Zinc	10 (9–13)	8 (7–11)	<0.001

Comparison between participants with normal and poor appetite: Mann–Whitney *U* test. A *P* value of <0.05 was considered statistically significant. Retinol activity equivalent (RAE), folate equivalent (FE).

Protein intake in relation to BW was also lower in participants with poor appetite (median 0.87 (0.69–1.06) g/kg BW) when compared with those with normal appetite (1.00 (0.80–1.21) g/kg BW, *P* < 0.001).

#### Intake of food groups

Daily intake of food groups in participants with normal and poor appetite is presented in Table 3. When compared with the participants with normal appetite, those with poor appetite had a lower intake of bread, legumes, vegetables, cheese, total meat and meat products, fish and shellfish, and alcoholic beverages and a higher intake of sugar and confectionery.

**Table 3:** Food group intake [g per day] and diet quality indices of the 569 participants aged 70 years and older of the Nutrition and Food-related Behaviour ancillary study of the LASA, stratified by appetite (median and quartiles 1–3)

Variable	Normal appetite (*n* = 498)	Poor appetite (*n* = 71)	*P* value
**Food groups**			
Rice, pasta	25 (10–49)	24 (9–50)	0.802
Bread	115 (82–48)	87 (70–141)	0.005
Potatoes, other tubers	84 (45–129)	63 (46–121)	0.099
Legumes	8 (0–19)	5 (0–13)	0.035
Vegetables	147 (101–201)	89 (50–118)	<0.001
Fruits	221 (107–273)	167 (82–271)	0.546
Dairy products except cheese	280 (157–428)	271 (137–429)	0.590
Cheese	29 (16–47)	20 (8–44)	0.005
Eggs	14 (7–22)	9 (5–17)	0.317
Total meat, meat products	74 (49–104)	57 (28–93)	0.001
Fish, shellfish	17 (8–30)	12 (8–20)	0.037
Sugar, confectionery	28 (13–48)	35 (15–63)	0.025
Cakes, sweet baked goods	38 (24–56)	42 (23–61)	0.425
Non-alcoholic beverages	1,308 (977–1,690)	1,310 (909–1,583)	0.429
Alcoholic beverages	63 (4–172)	2 (0–114)	<0.001
**Diet quality indices**			
DHD15	83 (73–93)	75 (68–83)	<0.001
MDS	33 (29–36)	29 (26–32)	<0.001

DHD15 range: 0–130; MDS range: 0–55; higher scores indicating higher adherence. Comparison between participants with normal and poor appetite: Mann–Whitney *U* test. A *P* value of <0.05 was considered statistically significant.

#### Quality of the diet

Both diet quality indices, MDS and DHD 15 were lower in participants with poor appetite than in those with normal appetite (Table 3).

### Associations between dietary characteristics and poor appetite

Poor appetite was associated with a lower intake of protein, folate, zinc and vegetables and a lower score of the diet quality indices DHD15 and MDS. Poor appetite was also associated with a higher intake of carbohydrates and vitamins B_2_ and C (Table 4).

**Table 4:** Associations between intake of energy, macronutrients, dietary fibre, alcohol, vitamins, minerals, food groups, dietary quality indices and appetite of 534 participants aged 70 years and older of the Nutrition and Food-related Behaviour ancillary study of the LASA

Logistic regression models	Odds ratio	95% confidence interval		*P* value
		Lower bound	Upper bound	
**Energy [kcal/d]**	1.000	0.999	1.000	0.200
**Macronutrients [g/d]**				
Carbohydrates	1.015	1.006	1.023	0.001*
Protein	0.948	0.922	0.973	<0.001*
Fat	0.992	0.971	1.013	0.460
Fibre	0.920	0.864	0.979	0.009
Alcohol	0.975	0.946	1.005	0.097
Water	0.999	0.999	1.000	0.039
**Vitamins**				
Vitamin A [μg RAE/d]	1.001	1.000	1.002	0.059
Vitamin B_1_ [mg/d]	1.067	0.190	5.980	0.941
Vitamin B_2_ [mg/d]	4.577	1.650	12.694	0.003*
Vitamin B_6_ [mg/d]	0.481	0.131	1.769	0.271
Vitamin B_12_ [μg/d]	0.690	0.522	0.912	0.009
Vitamin C [mg/d]	1.013	1.005	1.021	0.001*
Vitamin D [μg/d]	0.990	0.804	1.220	0.928
Folate [μg FE/d]	0.981	0.973	0.989	<0.001*
**Minerals [mg/d]**				
Calcium	1.001	0.999	1.003	0.243
Iron	0.982	0.714	1.351	0.912
Phosphorus	1.000	0.997	1.003	0.814
Magnesium	0.991	0.981	1.001	0.069
Zinc	0.619	0.454	0.846	0.003*
**Food groups [g/d]**				
Rice, pasta	1.004	0.997	1.012	0.270
Bread	0.993	0.985	1.000	0.062
Potatoes, other tubers	0.999	0.992	1.005	0.688
Legumes	0.990	0.968	1.014	0.422
Vegetables	0.988	0.982	0.994	<0.001*
Fruits	0.999	0.997	1.001	0.409
Dairy products except cheese	1.000	0.998	1.001	0.735
Cheese	0.990	0.978	1.003	0.138
Eggs	0.997	0.974	1.020	0.791
Total meat, meat products	0.986	0.975	0.996	0.007
Fish, shellfish	0.990	0.972	1.009	0.292
Sugar, confectionery	0.999	0.986	1.012	0.884
Cakes, sweet baked goods	1.001	0.989	1.014	0.813
Non-alcoholic beverages	1.000	0.999	1.000	0.570
Alcoholic beverages	0.998	0.995	1.001	0.212
**Diet quality indices**				
DHD15	0.964	0.945	0.983	<0.001*
MDS	0.904	0.850	0.961	0.001*

Binary logistic regression of dietary characteristics (reference category: normal appetite). Separate models were calculated for energy, each macronutrient, all micronutrients, all food groups, each diet quality index. Models are adjusted for sex, age, education, any involuntary weight loss in past 6 months, eating less because of chewing problems, polypharmacy, depression, energy intake (except for model with energy intake as independent variable). Normal appetite *n* = 472, poor appetite *n* = 62. ^*^A *P* value of <0.004 was considered statistically significant. Folate equivalent (FE), retinol activity equivalent (RAE).

## Discussion

In the current analysis of cross-sectional data from the LASA, 12.5% of the participating Dutch community-dwelling adults aged 70 years and older had poor appetite and showed poorer overall diet quality with lower scores of DHD15 and MDS when compared with those with normal appetite. Poor appetite was also associated with a lower intake of protein, folate, zinc and vegetables and a higher intake of carbohydrates, and vitamins B_2_ and C. There was no difference in energy intake between participants with normal and poor appetite.

The observed prevalence of 12.5% poor appetite is in the lower range of prevalence rates of 11– 27% reported in previous studies in community-dwelling older adults [2–4]. Differences in the observed prevalence rates may result from different methods of appetite assessment and different characteristics of the samples. There is no gold standard for assessing appetite in older adults [25], but the current analysis used SNAQ, a simple and easy-to-use tool, that shows robust predictive validity based on weight loss and malnutrition [14, 26]. Similarly, Hara *et al*. [10] used SNAQ with a cut-off of 14 to assess appetite. In contrast, van der Meij *et al*. [3] asked a single question on appetite in the past month and categorised the five answer options into very good, good and poor (moderate, poor, very poor) with very good as the reference category.

In line with van der Meij *et al*. [3], no association was found between poor appetite and energy intake in the present analysis. However, Hara *et al*. [10] reported an association with inadequate energy intake. One possible explanation for the missing association in the current analysis is that older adults with poor appetite may eat deliberately because they are aware that they need to eat. However, despite having comparable energy intake in the present analysis, diet quality was found to be poorer, reflected in lower scores of both the DHD15 and the MDS. Diet quality in relation to appetite has previously only been reported by van der Meij *et al*. [3], who found a significantly lower Healthy Eating Index [27] amongst participants with poor appetite in an unadjusted group comparison. Our findings do not support the hypothesis that poor appetite leads to energy malnutrition but rather indicate a reduced quality of nutrition. Energy intake may only be reduced in individuals with poor appetite who have a worse health status or acute health problems that trigger the development of malnutrition. Future studies should investigate this relationship further.

One of the most relevant factors concerning the prevention of malnutrition is protein intake. Poor appetite was associated with a lower protein intake, which is in line with previous findings [3, 10]. Protein intake was also lower when expressed per kg BW (median normal vs. poor appetite: 1.00 vs. 0.87 g/kg) and less favourable in those with poor appetite when compared with the recommended intake of at least 1.0 g/kg BW to maintain muscle mass, which may affect physical performance [28]. Participants with poor appetite in the present analysis presented a low SPPB more frequently in the unadjusted group comparison, which underlines this link. In older adults with long-term insufficient protein intake, adverse adaptive responses may occur, including a reduction in muscle size and function [29], whereas a higher protein intake is associated with higher muscle mass and reduced loss of lean mass in older adults in observational studies [30, 31]. A lower protein intake may therefore provide an important link between poor appetite and associated adverse outcomes such as disability [5] and mortality [6, 7].

It is worth noting that most nutrients were consumed within the recommended intake levels and there were no relevant deficiencies in the poor appetite group [32]. However, fibre intake was below the recommended amount of 25 g/d [32] in both appetite groups with significantly lower intake in the poor appetite group. The intake of vitamin D was also very low in both groups; however, vitamin supplements were not included in the analyses.

Poor appetite was also associated with a lower intake of folate and vegetables. Vegetables are an important source of folate [17]. Thus, the lower intake of vegetables may explain the lower folate intake. In line with these findings, van der Meij *et al*. [3] reported a lower intake of vegetables in participants with poor appetite. In the current analysis, poor appetite was associated with a lower intake of zinc, the insufficient intake of which was also associated with poor appetite in the analyses from Hara *et al*. [10]. Research indicates that zinc deficiency potentially influences nutritional intake by a decline in taste, which can reduce appetite [33]. We cannot explain the association of poor appetite with a higher intake of vitamin C, especially as the intake of vegetables was reduced. The effect size for the association between poor appetite and vitamin B_2_ intake is characterised by a very large confidence interval (Table 4) and should be interpreted with caution. Whilst this analysis revealed a lower intake of dietary fibre only in the unadjusted group comparison, other studies found an association between poor appetite and a lower intake [3] or inadequate intake [10] of fibre in multivariate analyses.

### Strengths and limitations

The present analysis had notable strengths. It was based on a large data set with data on many characteristics from different domains of life, which were used to adjust the models for potential confounding. The LASA ancillary study on Nutrition and Food-related Behaviour comprehensively assessed dietary intake, using a validated FFQ, which allowed the consideration of a large number of dietary characteristics [34]. Appetite was assessed using the standardised and validated questionnaire SNAQ [14], providing a good basis for comparison with previous and future studies on this topic. This is particularly important as there is no gold standard for assessing appetite in older adults and many different assessment methods are being used [25].

Several limitations of this study should also be mentioned. The results were based on a Dutch sample of older adults and cannot be generalised to older adults from other countries because of possible differences in food preferences and dietary patterns. The cross-sectional design does not allow any conclusions about cause and effect. The general characteristics were obtained in a different year prior to the dietary characteristics. Three participants were excluded from the analysis because of implausibly low energy intake, one of whom would have been assigned to the poor appetite group and may actually have consumed so little energy. Because of the relatively low prevalence of poor appetite, we were not able to include all dietary characteristics in a single regression model and were therefore unable to incorporate a possible confounding or interaction between the explanatory variables. This could be addressed in future analyses by utilising larger data sets.

## Conclusion

In this sample of Dutch community-dwelling older adults, poor appetite was associated with lower diet quality scores, lower protein, folate, zinc and vegetable intake as well as higher carbohydrate, and vitamins B_2_ and C intake, whereas energy intake was not different. These results reflect a more unfavourable dietary pattern and a lower overall diet quality associated with poor appetite. Dietary counselling in older adults with poor appetite may help to improve diet quality and reduce the future risk of developing malnutrition and diet-related diseases.

## Data Availability

Data from the LASA are available for use for specific research questions, provided that an agreement is made up. Research proposals should be submitted to the LASA Steering Group, using a standard analysis proposal form that can be obtained from the LASA website: www.lasa-vu.nl. Files with data published in this publication are freely available for replication purposes and can be obtained using the same analysis proposal form. The LASA Steering Group will review all requests for data to ensure that proposals for the use of LASA data do not violate privacy regulations and are in keeping with informed consent that is provided by all LASA participants.
